# Preparation and reverse recycling logistics of a new type of nano-filled antibacterial layer packaging film for dairy products

**DOI:** 10.3389/fchem.2023.1302198

**Published:** 2023-12-12

**Authors:** Quan Li, Xiaorong Zhou, Hong Wu

**Affiliations:** ^1^ Department of Art, Nanchong Vocational and Technical College, Nanchong, Sichuan, China

**Keywords:** dairy product packaging film, nano-filling, antibacterial layer, reverse recycling logistics, food safety

## Abstract

**Introduction:** Dairy products are loved by people because of their high nutritional value, but they have also become the most ideal breeding places for microorganisms. Some dairy packaging has the problem of lax sealing, resulting in products susceptible to contamination and deterioration. The harmful microorganisms and bacteria contained in them will pose a serious threat to people’s health. Therefore, a good antibacterial protection is very important for dairy products. The purpose of this paper is to study the preparation and reverse recycling logistics of a new type of nano-filled antibacterial layer packaging film for dairy products.

**Methods:** A new type of nano-filled antibacterial layer packaging film is prepared by extrusion casting method, and its mechanical properties and antibacterial properties are analyzed.

**Results:** The experimental results in this article show that the prepared new nano-filled antibacterial layer packaging film has lower light transmittance and water vapor transmission rate, and has obvious antibacterial properties against *Staphylococcus aureus* and *Escherichia coli*, and has good barrier properties.

**Discussion:** The antibacterial rate of the bacteria in the petri dish is as high as 99.97% after being placed for 120 days, and the antibacterial performance can be enhanced by the ratio of glycerol and starch content, and the new nano-filled antibacterial film prepared is degradable Sex, can be better recycled.

## 1 Introduction

Dairy products are rich in nutrients and contain a variety of nutrients such as protein. They are one of the main sources of protein nutrition for people and an ideal medium for various microbial floras. Because dairy products are perishable, how to maintain the original quality and characteristics of dairy products and avoid the influence of oxygen, microorganisms, temperature, humidity and other factors has always been an important issue. Most ready-made dairy products, such as pasteurized milk and yogurt, should be stored at low temperatures to maintain good quality and nutritional value. However, low-temperature storage does not inhibit all microorganisms, such as cold-eating bacteria. According to the literature, about 25% of dairy product life problems are caused by heat-resistant psychotropic bacteria. Other common microorganisms, such as yeasts and molds (Geochophyllum, Fire mold, Mucor, *Mycoplasma*, Penicillium) can cause significant changes in taste, texture, and appearance. In addition, the development of heat-resistant lactococcus can lower the pH of pasteurized milk until coagulation occurs. This article aims to prepare a new type of antibacterial layer packaging for the antibacterial of dairy products to improve the antibacterial ability of dairy products and reduce the safety problems of dairy products.

With the improvement of people’s living standards and environmental protection awareness, new functional materials and antibacterial materials are becoming more and more common in various fields of human production and life. So far, antibacterial materials have been used in many fields such as medical treatment, health/equipment, building materials, food packaging and so on. The isolation and maintenance of bacteria is also an important link in the storage and sales of dairy products, and it is also related to the health of consumers. Bacteriophages have always been a hot topic in the storage and storage of dairy products. Therefore, the antibacterial research of dairy products is particularly important.The nano-antibacterial layer packaging film has excellent antibacterial properties, which can effectively inhibit the growth of bacteria and other microorganisms, thereby extending the shelf life of dairy products.

Different polymer films and different headspace conditions (air, vacuum, and four different modified atmosphere packaging) are combined to select a packaging system to ensure two portions of Canestrato mature for 4 months and 12 months the shelf life of di Moliterno dairy products. In order to evaluate the quality deterioration of packaged dairy product samples under refrigerated conditions, Costa C monitors texture changes, weight loss, water activity, moisture content, microbial contamination, pH and sensory properties during storage. The results show that the high-barrier multilayer film delays the proliferation of mold and provides the best effect for the two types of dairy products (Costa et al., 2016). Lim J Y determined the efficacy of using lactic acid bacteria and grapefruit seed extract (GSE) packaging to control the growth of *Listeria* monocytogenes in soft cheese. Leuconostoc mesenteroides and *Lactobacillus campylobacter* isolated from kimchi are used as starters to make soft cheese and inoculated with cocktail strains of *Listeria* monocytogenes. Dairy products are packaged with low-density polyethylene, biodegradable polybutylene adipate-butylene terephthalate (PBAT), low-density polyethylene and GSE or PBAT and GSE, and packed at 10°C and store at 15°C. Leuconostoc mesenteroides (LcM) inhibited the growth of *Listeria* monocytogenes better than Lb. Curvature. PBAT with GSE film showed the best control over the growth of *Listeria* monocytogenes. When both LcM and PBAT and GSE are applied to soft cheese, the growth of *Listeria* monocytogenes is significantly more inhibited than using LcM or PBAT and GSE alone (Lim et al., 2020). Rajendran A has successfully grown a nanostructured titanate layer encapsulated with Ca2+ and Ag + ions on commercially pure (CP) Ti metal by chemical treatment with H2O2 and subsequent treatment with Ca(NO3)(2)/AgNO3 solution. The nanostructured titanate layer is further transformed into titanium dioxide containing Ca2 + and Ag + ions. When immersed in simulated body fluid (SBF), the modified titanium metal showed a significant increase in apatite-forming ability. The presence of Ag + ions shows good antibacterial activity against pathogenic *S. aureus*. The ratio of Ca2 + and Ag + ions on the surface of titanium metal can be optimized to obtain the lowest Ag concentration. This concentration not only has antibacterial activity, but also can be compared with MG 63 is compatible with osteoblast-like cells (Rajendran et al., 2019). Zhou H used a magnetron sputtering device to design and deposit a multifunctional VO2/ZnO double-layer film. Combining anti-reflection, anti-oxidation and anti-corrosion functions with anti-bacterial properties makes heterostructure films a promising candidate for energy-saving smart windows. ZnO film as an anti-reflection layer can significantly increase the solar conditioning efficiency (ΔTsol) from 7.7% to 12.2%, and has excellent light transmittance in the low-temperature semiconductor phase (Tlum-L = 50.3%). As a protective barrier, the ZnO layer can not only protect the VO2 film from being oxidized into highly toxic V2O5, but also reduce the release of V ions. In addition, the synergistic effect of Zn2+ ion release killing effect and ZnO nanoparticle contact killing effect makes ZnO film an excellent antibacterial coating. In terms of biological safety, a ZnO coating with an appropriate film thickness can effectively reduce the cytotoxicity of VO2 to human HIBEpiC cells. We hope that this work can provide new insights for the better design of new multifunctional VO2-based smart energy sources (Zhou et al., 2016). In this study, Lee B S synthesized a nanocomposite coating composed of polydopamine, functionalized poly (3, 4-ethylenedioxythiophene) (PEDOT) and silver nanoparticles (AgNPs) by layer-by-layer deposition. Biomimetic polydopamine and hydroxyl functionalized PEDOT are used to enhance adhesion strength. The deposition of PEDOT functionalized with zwitterionic phosphorylcholine contributes to antifouling properties. After being immersed in the AgNO3 solution, Ag + ions are adsorbed on the PEDOT film and further spontaneously reduce to form AgNPs, thereby giving these nanocomposite films antibacterial properties. *Escherichia coli* and *Streptococcus* mutans were chosen to represent two common Gram-negative and Gram-positive oral pathogens. Inductively coupled plasma mass spectrometry was further carried out to confirm that the Ag + ions released from these nanocomposite films will not have an adverse effect on the human body (Lee et al., 2019). Baysal G studied the preparation of quaternary ammonium salt modified montmorillonite such as cetyltrimethylammonium bromide (CT), cetyltributylphosphonium bromide (HD) and corn starch (CS) for food Antibacterial nanocomposite film for packaging. After that, it confirmed the antibacterial activity of CS nanomembrane against *S. aureus* and *E. coli* bacteria. X-ray diffraction (XRD), Fourier transform infrared spectroscopy (FT-IR) and scanning electron microscopy (SEM) were used to analyze the dispersibility of the silicate layer and the starch nanocomposite film. The results showed that the presence of quaternary ammonium salt enhanced the dispersibility of clay and starch film combined with quaternary ammonium salt will provide potential applications in food packaging as a nanostructured material. The nanomembrane obtained according to the results of antibacterial analysis was confirmed to have stronger antibacterial properties than similar studies in the literature (Baysal and Celik, 2019). The test methods, conditions and standards used in different scholars’ studies are different, resulting in poor consistency and comparability of data. This makes it difficult to compare and analyze the results of different studies, which limits the further promotion and application of the research.

The innovation of this article is to study the nano particles of different types of antibacterial agents. Compared with the current starch film with antibacterial properties by adding one or several antibacterial agents directly to the starch solution, the antibacterial packaging film prepared in this article has In addition to good antibacterial properties, the mechanical properties are also good, the light transmittance and water vapor transmission rate are also well maintained, and it has good biodegradability, safety and non-toxicity, and meets the requirements of environmental protection and safety.

## 2 Preparation method of nano-filled antibacterial layer packaging film for dairy products

### 2.1 Nano-filled antibacterial layer packaging film

Antibacterial food packaging film is a functional film with the ability to inhibit or kill surface bacteria. According to the form of antibacterial agents, it can be divided into direct antibacterial agents and indirect antibacterial agents (Park et al., 2018). Direct antibacterial agent is the direct addition of antibacterial ingredients to packaging materials that are in direct contact with food. Indirect antibacterial agents are the addition of certain substances, which can control the growth of microorganisms by adapting the microenvironment of food packaging to the carrier or using the selective permeability of the material (Kazarian, 2017).The morphological difference between direct and indirect antimicrobials is that direct antimicrobials are mainly in solid form, while indirect antimicrobials are mainly in liquid form. Nano-composite food packaging film is a composite film material formed by embedding nano-level components in various substrates, which has the advantages of traditional composite materials and modern nano-materials. Due to the surface effect, volume effect, size effect and other characteristics of the special structure of the composite film, nano has characteristics that conventional materials do not have, such as optical properties, mechanical properties, antibacterial properties, barrier properties, etc. It has a wide range of uses in packaging (Forcinio, , 2017; Okano, 2017). Food packaging films made from a single natural polymer biomaterial still have major defects in their mechanical properties, mechanical properties, and anti-activity. Therefore, researchers often modify the natural polymer materials based on their own defects before using them. As a food packaging raw material, food packaging films with better performance have been obtained. For example, adding nano-oxides, according to the structural characteristics of nano-oxides, is easy to enter the cell membrane, which can destroy the bacterial structure and achieve the purpose of antibacterial; adding essential oils can effectively improve the antibacterial properties of the membrane (Co et al., 2017; Kazemzadeh et al., 2017).

### 2.2 Preparation method of nano-filled antibacterial layer packaging film for dairy products

The same film-forming solution and different film-forming processes produce different film properties. At present, there are many film-forming processes, such as layer-by-layer self-assembly film formation, sol-gel method, *in-situ* polymerization method, etc. (Roy et al., 2017; Palanisamy et al., 2018). However, the methods commonly used in laboratories mainly include solution casting method, extrusion casting method and electrostatic spinning technology (Hu and Wang, 2016; Rokaityte et al., 2016).

(1) Solution casting method

Solution casting method is currently the most commonly used film forming technology in laboratories. The nanocomposite film for food packaging was prepared by the solution flow method, and Fourier infrared and scanning electron microscopy were used to characterize the film structure (Daman et al., 2018). Scanning electron microscopy shows that nano-silver oxide particles are uniformly distributed on the entire surface of the film; the addition of nano-zinc oxide can significantly reduce the oxygen permeability and heat sealability of the film; ultraviolet and infrared indicate that the absorbance of the film is significantly affected by nano-zinc oxide (Aviat et al., 2016).

(2) Extrusion casting method

Extrusion casting method refers to uniformly adding the film-forming substrate to the screw extruder at a constant feeding speed, controlling the temperature of the barrel and the head, adjusting the size of the film outlet gap and the rotation frequency of the traction roller to obtain continuous and smooth Packaging film (Qi et al., 2020).

(3) Electrospinning technology

Electrospinning is a technique for preparing nanofibers with the help of high-voltage electrostatic field force (LiuYaowenWang et al., 2018). Its main working principle is that the polymer solution is charged by the high-voltage electrostatic field force to form cone-shaped droplets or “Taylor cones” at the tip of the nozzle. At this time, the droplet is subjected to the dual action of its own surface tension and the repulsive force of the surface charge caused by the electrostatic field. When the charge repulsive force on the surface of the droplet exceeds its surface tension, a small stream of polymer liquid is sprayed onto the surface of the solution at a high speed. The flow of liquid is stretched and pulled at high speed in a short distance under the action of electric field force, so that the solvent evaporates and solidifies, and finally deposits on the receiving plate to form polymer fibers (Jalil et al., 2016).

Solution casting method, extrusion casting method and electrospinning technology are common materials processing methods.

The advantage of solution casting is that materials with complex shapes can be prepared, and the microstructure and properties of the materials can be controlled. The disadvantage is that the production cost is higher, and the requirements for raw materials and equipment are higher.

Extrusion casting method has the advantage of high production efficiency, can continuously produce a large number of products; The disadvantage is that the requirements for raw materials are high, and the shape of the product is limited.

The advantage of electrospinning is that fibrous materials can be prepared and the microstructure and properties of materials can be controlled. The disadvantage is that the production efficiency is low, and the equipment and operation requirements are higher.

### 2.3 Research methods of the performance of antibacterial protective film

In the basic process of the shape profile, it will pass through the shear flow field, and the melt will be elastically deformed. After the die is exited, the molecules will return to the original shape to a certain extent, causing the phenomenon of extrusion growth (Kochan et al., 2016; Roudbari et al., 2021). The squeeze out will cause body entanglement problems, which will affect the quality control of the final product (O'Reilly and Kumar, 2016; Guo and Zhang, 2017). The factors that affect the growth of the compound rubber extrusion include temperature, shear speed record, filler and molecular weight, which can be suppressed by changing the temperature, shear rate, die design, additives and other methods in the production process (Demirel et al., 2016).

In this paper, the pressure loss value of the capillary inlet is calculated by the pressure sensor. The calculation is as follows:

$$G_{0}=G_{a}-\frac{g_{b-}g_{a}}{b_{i}-b_{a}}b_{a}$$ (1)

Since there is a zero-length die for comparison, it is very convenient to calibrate the inlet pressure, and there is no need to perform Bagley calibration. Assuming that the cross-sectional area of the barrel is s, the plunger velocity is p, the radius of the capillary is r, and the power law exponent of the melt is m, the calculation formula for the related physical quantity is as follows:

$$\kappa_{a}=\left(\frac{2m+1}{2m}\right)\frac{3Q}{\pi r^{2}}\prod v_{m}$$ (2)

$$\varpi =\frac{\left(G_{a}-G_{b}\right)r+\left(G_{a}-G_{b}\right)v}{2b_{a}}$$ (3)

$$\nu_{a}=\frac{\varpi }{{\dot{\kappa }}_{a}}s\left(v_{m}\frac{3Q}{\pi r^{2}}\right)\left(\frac{2m+1}{\varpi }\right)$$ (4)

These two sensors can also monitor changes in melting pressure from time to time to check the stability of the extrusion process. By analyzing the shear viscosity effect of the rubber material measured by the capillary flowmeter, the influence of temperature, shear rate and other factors on the molten rubber material can be investigated to guide actual production.

When a normal sine wave voltage is applied to the sample, the sample will feed back a normal sine wave voltage. There will be a phase difference η between two sine waves, η is generally between 0°–90°, the ratio of stress amplitude to strain amplitude can be calculated as:

$${\bar{W}}_{i}=\left|w*\right|\cos\eta \frac{g\eta_{i}}{g\eta_{j}}wg$$ (5)

$${\bar{W}}_{j}=\left|w*\right|sin\eta \frac{g\eta_{j}}{g\eta_{i}}wg$$ (6)

Among them, ${\bar{W}}_{i}$ is the storage modulus, which means the elastic part, which is the part stored in the deformation energy, and ${\bar{W}}_{j}$ is the stored modulus, which means the viscous part, which is the part lost in the deformability.

$$P_{ij}=\frac{\left(X+Y\right)*\left(a/2\right)}{P_{i}\times P_{j}}$$ (7)

Among them, X and Y are constants related to the system. After reaching the stable period, the performance of the rubber compound remains basically constant. According to the change of the combined glue amount of the filler during the parking process, a modified relationship is proposed:

$$XP_{a}=xp_{0}+\left(xp_{l}-xp_{0}\right)\left(1-\exp\left(-pa^{\frac{1}{2}}\right)\right)$$ (8)

### 2.4 Nano-filled drug release behavior

In specific *in vitro* drug release studies, drug release methods with different carriers should be used rationally. So far, the mathematical models used in drug release studies can be roughly divided into six types (Zhang et al., 2023; Zhao et al., 2023). Polynomial models, such as dynamic model, it was probability distribution model, Gompertz model, accounting model, second-order, third-order and higher-order polynomial models. (6) Other models, such as Higuchi model and Hixon Crowell model. Among them, there are three commonly used.

(1) Zero-order dynamics model expression:

$$R=R_{i}\frac{Ke_{i}}{\left(s_{0}-s_{1}\right)}s_{0}<s<s_{1}$$ (9)

Where R is the cumulative percentage of the drug in the solvent when the time is s, $R_{i}$ represents the initial drug concentration in the solvent, $e_{i}$ is the zero-order release constant, and s represents the release time.

The zero-level model expression based on the COD model:

$$R=R_{i}\frac{Ke_{i}\times \left(1-\varepsilon \right)\times Z\times COD}{\left(s_{0}-s_{1}\right)}s_{0}<s<s_{1}$$ (10)

The expression of the zero-level model based on the generalized molecular model is:

$$R=R_{i}\frac{Z\left(1-\varepsilon \right)t_{0}}{\left(s_{0}-s_{1}\right)}s_{0}<s<s_{1}$$ (11)

Zero-level model based on biodegradable model:

$$R=R_{i}\frac{\left(1-\varepsilon \right)\times z\omega }{\left(s_{0}-s_{1}\right)}s_{0}<s<s_{1}$$ (12)

(2) First-order dynamic model expression

$$R=R_{l}\left(1-\frac{G}{R}\right)e^{-k_{i}s}\left(1-\frac{r}{k}\right)e$$ (13)

In the formula, $R_{i}$ is the maximum cumulative release degree, G is a constant, and $K_{i}$ is the first-order release constant.

(1) *In vitro* release of nano-microspheres

Use natural antibacterial agents to make nano-microspheres through a certain amount of proportioning and emulsification and cross-linking method, and calculate the *in vitro* release of nano-microspheres. $T_{0}$ is the release percentage, and the calculation formula is:

$$T_{0}=\frac{B_{n}\times Q+Q\sum_{n=0}^{i-1}B_{i}}{W\times G_{l}\%}\times 100\%$$ (14)

Where $B_{n},B_{i}$ is the concentration of the samples taken at the nth and i-th time points; Q is the total volume of PBS used, and W is the total mass of the microspheres taken in the experiment. $G_{l}\%$ is the drug loading rate of the drug-loaded microspheres.

### 2.5 Performance characterization of new nano-filled antibacterial layer packaging film for dairy products

(1) Water vapor transmission rate detection

The prepared new nano-filled antibacterial layer packaging film was dried and tested for water vapor transmission rate. Using the pseudo-cup method, put the flowing out CaCl2 into the last beaker at 25°C, and let the added CaCl2 reach the mouth of the cup At 5 mm. Choose a uniform non-porous film, measure its thickness with a micrometer, put it in a cup, seal it with paraffin and weigh it. Place the weighed glass in a bottom deionized water dryer at 25°C (maintain 100% relative humidity) to keep the vapor pressure difference between the inside and outside of the membrane constant, and take out the glass regularly (Zhang et al., 2020). And calculate the water vapor transmission coefficient according to the formula, and the formula is as follows:

$$WVP=n\frac{\nabla n\times b}{S\times \nabla a\times \nabla b}\times \frac{2\nabla b+\nabla n}{\nabla a}$$ (15)

In the formula, WVP represents the water vapor transmission coefficient, n represents the increase in mass, S represents the area of the film, b represents the test time interval, and a represents the thickness of the film.

(2) Light transmittance detection

The calculation formula of light transmittance is:

$$L_{i}=\mathrm{lg}\left(\frac{1}{i}\right)=\mathrm{lg}\left(\frac{l_{0}}{l_{j}}\right)$$ (16)

L represents light intensity. The greater the light intensity absorbed by the film, the smaller the intensity of the transmitted light. $D=\left(\frac{l_{j}}{l_{j}}\right)$ represents the light transmittance, and the degree of reflection is represented by the reflectance S.

$$S={\left(n-1\right)}^{2}/{\left(n+1\right)}^{2}\times 100\%$$ (17)

Among them, n is the refractive index of the test control material. The smaller the reflectivity was the greater the light transmittance and the better the transparency.

(3) Transparency detection

The transparency is measured by an ultraviolet spectrophotometer. The film is cut into 20*20 mm squares and pasted on the surface of the cuvette. Using the blank cuvette as a blank reference, the absorbance of the film is measured, and the transparency of the film is calculated as:

$$P=\frac{-\log P_{l_{0}}}{l_{i}}\sum_{i}^{p}\left(-\log P\right)$$ (18)

Where P represents the light transmittance of the sample film, and $l_{0}$ represents the thickness of the film. It can be known from this formula that the higher the value of the light transmittance P, the lower the transparency of the representative film.

(4) Mechanical performance calculation

A physical property tester was used to determine the tensile strength and elongation at break. Under the influence of axial tension, the ratio of the maximum tensile load to the product of the width and thickness of the film before rupture is calculated as follows:

$$Q_{l_{0}}=\frac{H_{l_{0}}}{l_{0}\times w_{0}}\int Q\frac{h}{lw}$$ (19)

Q represents the tensile strength, H represents the maximum tension at break, and w represents the thickness of the film.

The formula for calculating elongation at break is:

$$K_{h}=\left(\frac{l_{i}\times w_{i}}{t}\right)\left(\frac{t_{l_{0}}-d_{l_{0}}}{d}\right)\times 100\%$$ (20)

## 3 Preparation experiment of new type nano-filled antibacterial layer packaging film for dairy products

### 3.1 Experimental materials

The materials used in the experiment are shown in Table 1:

**TABLE 1:** Laboratory reagents and drugs.

Number	Drug name	Purity
1	glycerin	\
2	starch	\
3	Span80	Chemically pure
4	Tween80	Analytically pure
5	Cacl_2_	85%
6	Na_2_S_2_O_2_	Analytically pure
7	NaOH	Chemically pure
8	Sodium Alginate	Chemically pure
9	Potassium Sorbate	Analytically pure
10	TiO_2_	Analytically pure

Table 1 shows 10 materials used for the preparation of the novel nano-filled antibacterial layer packaging film for dairy products, among which Span80, NaOH, Sodium Alginate are chemically pure, while Tween80, Na_2_S_2_O_2_a, Potassium Sorbate are analytically pure.

### 3.2 Preparation by extrusion casting method

**FIGURE 1:**
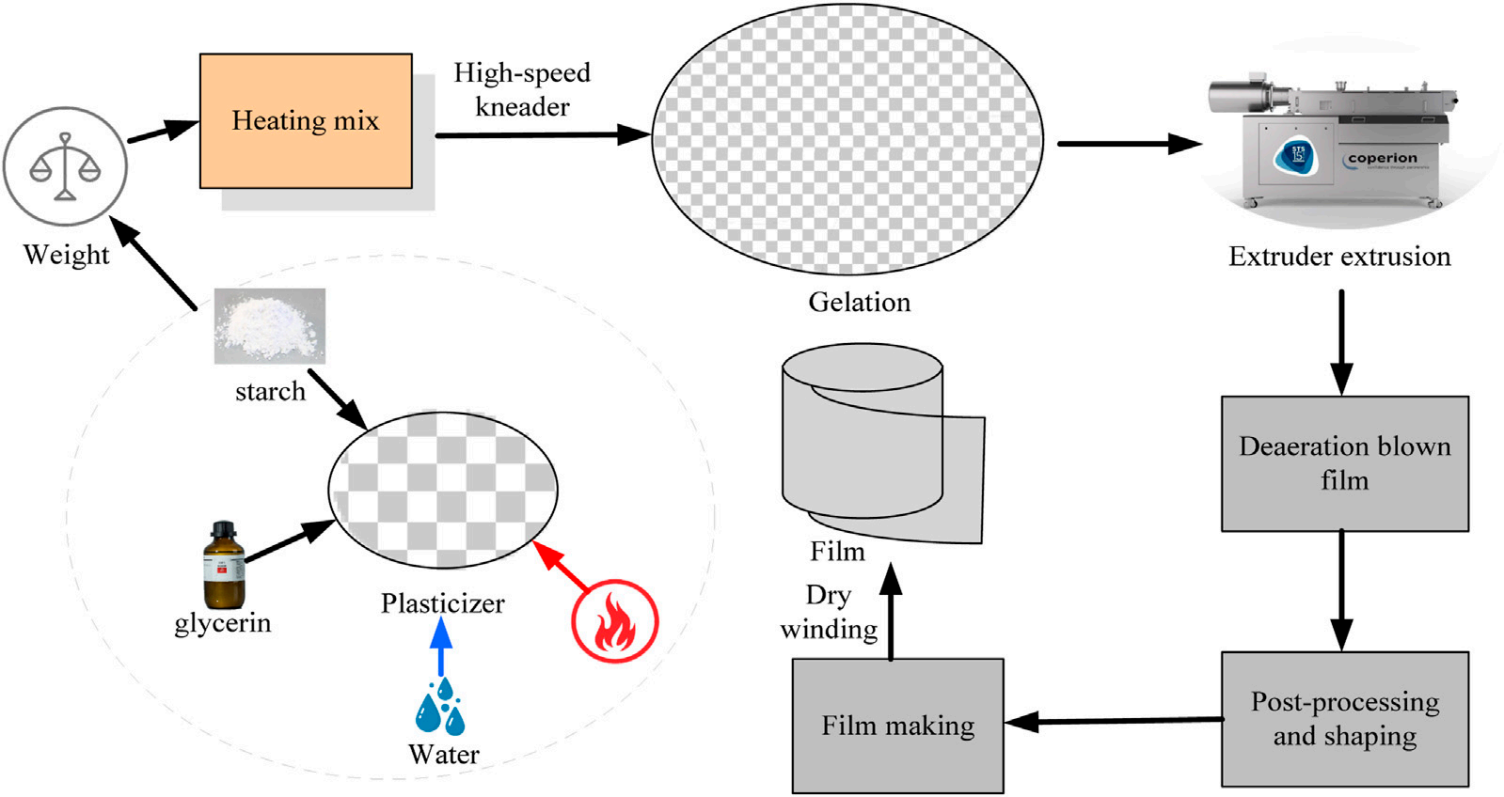
Extrusion casting method experimental preparation process.

The extrusion casting method is divided into two-stage extrusion and one-stage extrusion. Primary extrusion means that the raw material is simply formed into a film through an extruder, but the starch and glycerin must be mixed before the extrusion can be mixed with the mixer. The manufacturing process is shown in Figure 1.

The main advantages of the extrusion casting method are relatively simple equipment and high construction efficiency. The final product is cylindrical and easy to prepare. The main disadvantage is that the film looks poor and requires a lot of vertical space to dry with a hair dryer. The manufacturing process involves several hours of drying (vacuum), rapid additive mixing, improved single ball screw extrusion using an extruder, secondary blow molding and post-processed film preparation, and step-by-step preparation.

(2) Preparation by solution casting method

**FIGURE 2:**
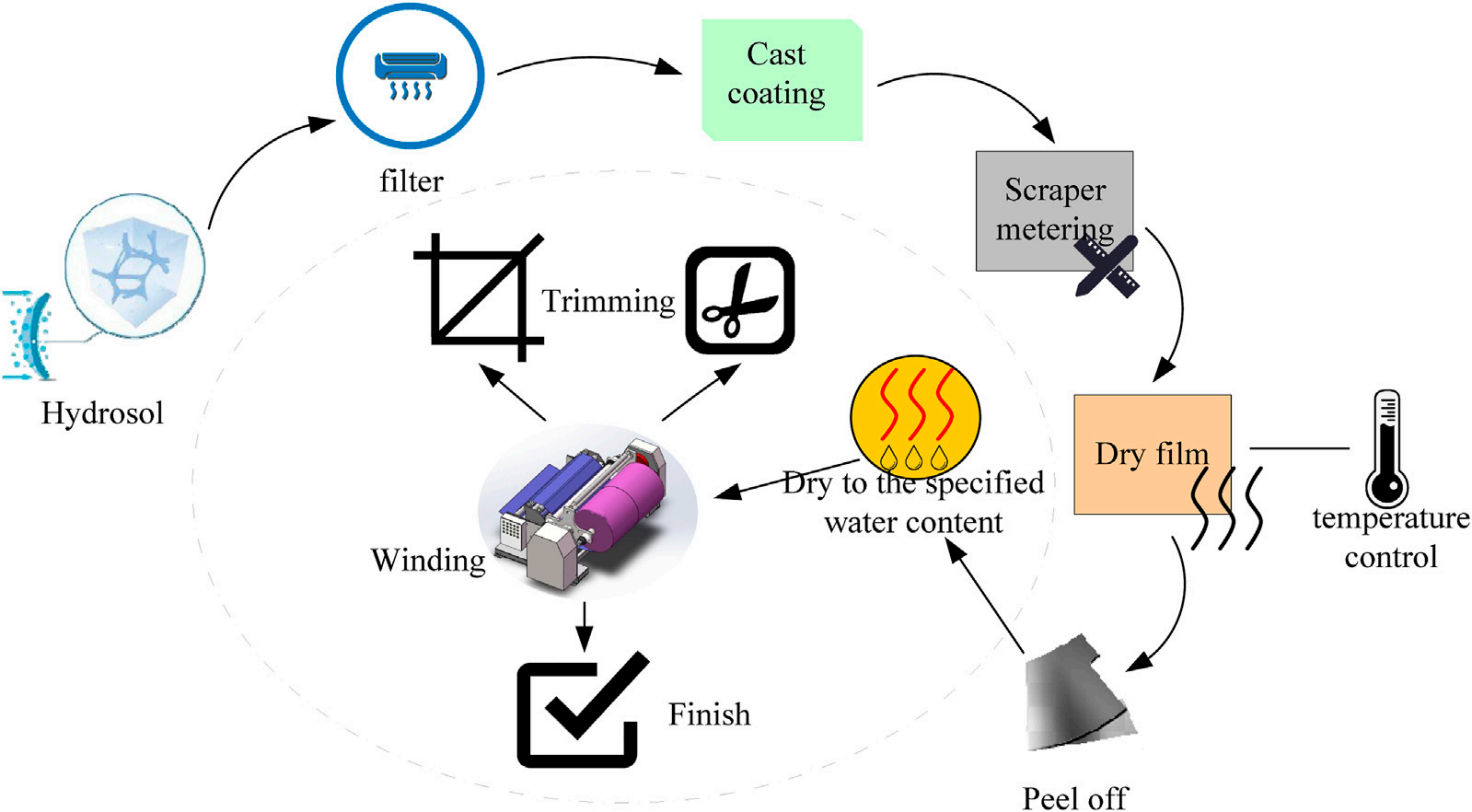
Solution casting method preparation process.

The solution casting method is to prepare a low-concentration aqueous solution and add corresponding additives (plasticizers, dyes, mold release agents, etc.) to make the solution form a steel strip or steel. By slit coating, the machine is coated on the coating series or belt, and the film is dried after heating (infrared heating, hot air heating or heating inside the film forming device). The manufacturing process is shown in Figure 2.

## 4 Mechanical properties and antibacterial properties of nano-filled antibacterial layer packaging film for dairy products

### 4.1 Performance analysis of the new nano-filled antibacterial layer packaging film

(1) Tensile resistance

**FIGURE 3:**
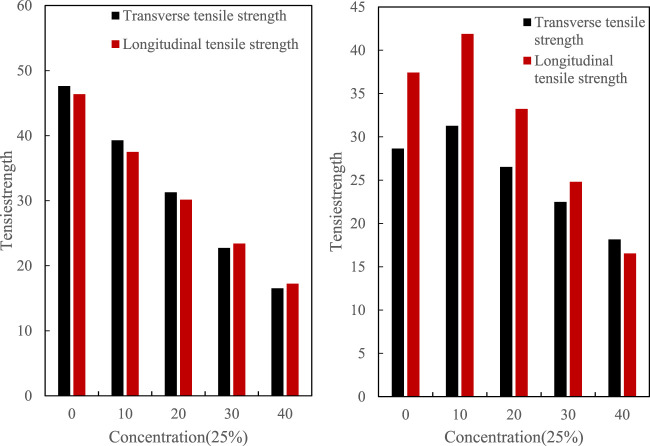
Tensile strength of 25% concentration film sample.

As a material, the most important performance is its mechanical properties, especially as a packaging material, tensile properties are more important. The tensile strength of dairy packaging film is closely related to packaging technology, packaging efficiency and material utilization rate. Suitable tensile strength can ensure that the packaging film is not easy to break or deformation during the packaging process, improve packaging efficiency, reduce waste and loss in the packaging process, reduce production costs, and improve economic performance.The tensile test of the film mainly monitors the tensile strength and elongation at break of the film in different directions in the transverse and longitudinal directions. The test of tensile strength can characterize the rupture of the film sample due to external force during transportation. The tensile strength test result is shown in Figure 3.

Figure 3 shows the tensile strength values ranging from 15 to 50 for both transverse and longitudinal ranges.

**FIGURE 4:**
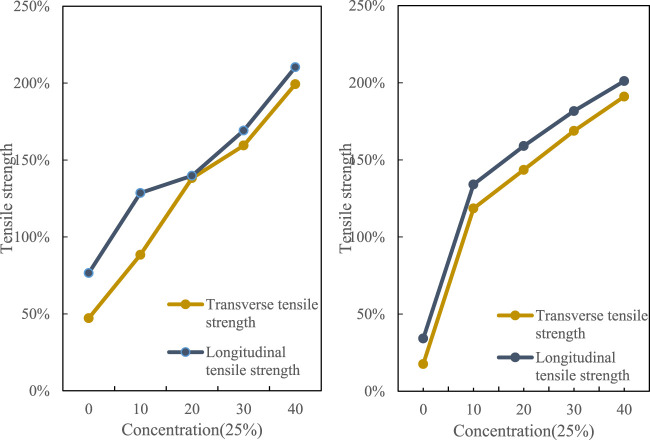
Tensile strength of 25% concentration film sample.

The final test result of elongation at break is shown in Figure 4.

It can be seen from the curve of the elongation at break of the film sample in the figure that with the increase of the dose, the toughness of the film sample also increases. Taking into account comprehensively, it may be due to the summary of the casting process and the adjustment of the temperature of the solution that the molecules are affected. The applied force produces an orientation arrangement along the longitudinal direction, which makes the sample anisotropy as a whole.

**FIGURE 5:**
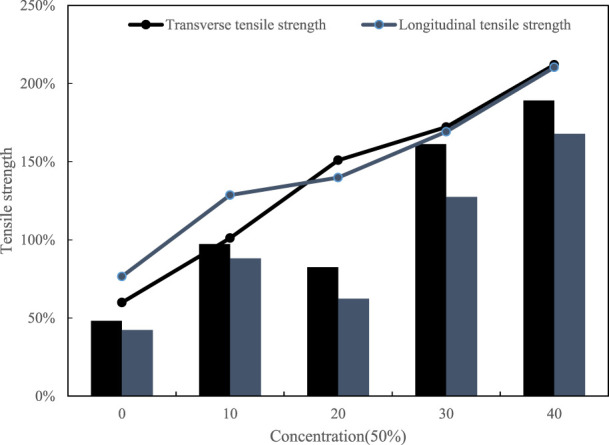
Tensile strength of 50% concentration film sample.

Figure 5 is a film sample with a concentration of 50%. The greater the concentration of the sol was higher the viscosity. At the same time, there will be strong hydrogen bonding between the macromolecular chains. Due to the excessive viscosity and the interaction between molecules, the crystallinity of the molecules is increased, which leads to an increase in tensile strength, improves the mobility of macromolecules, and changes the tensile strength.

(2) Analysis of light transmittance

**FIGURE 6:**
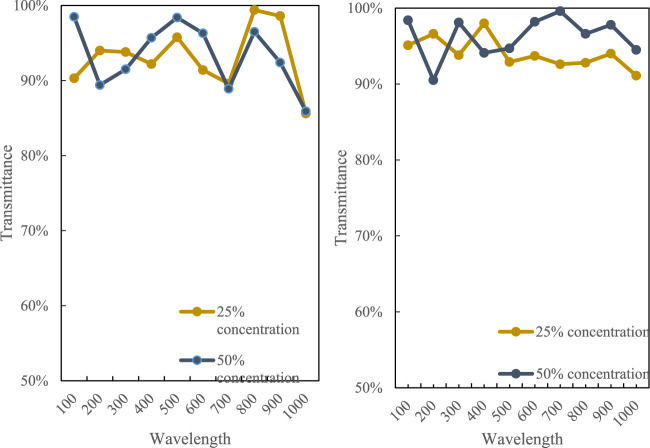
Light transmittance of film samples at different wavelengths.

The film has a very high degree of crystallinity of starch molecules, so its light transmittance is relatively low. Before the film sample is tested for light transmittance, a section of the sample is selected for full-wavelength scanning. The results are shown in Figure 6:

It can be seen from the figure that the light transmittance of the film sample is related to the wavelength. The light transmittance of the film sample is at a lower position. The light transmittance of the film sample with a solution concentration of 25% is 85%–99.5%, 50% the light transmittance of the film at the solution concentration is 90%–99%. Relatively speaking, as the concentration of the solution increases, the molecular crystallinity of the film will also increase, so the light transmittance decreases.

(3) Water vapor transmission rate

**FIGURE 7:**
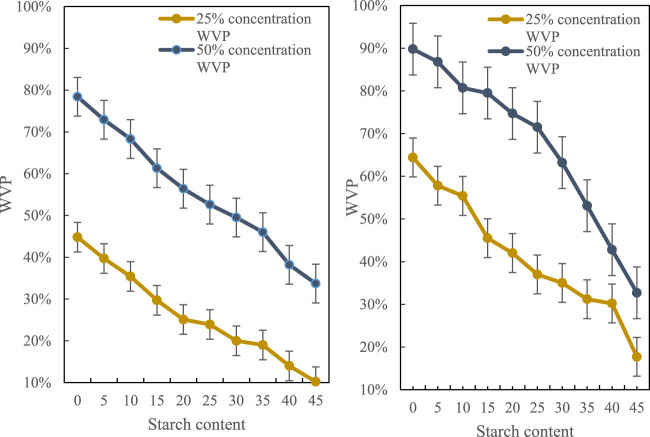
Test results of water vapor transmission coefficient of film samples.

The prepared film has slight oil permeability, indicating that the barrier to oil is still considerable. Starch is added in the film preparation. The starch molecule contains a large number of hydroxyl groups and has good compatibility with water molecules. The film is added to starch. The experimental results of the water vapor transmission coefficient (WVP) are shown in Figure 7:

It can be seen from the figure that the added starch dosage will decrease the water vapor transmission rate as the dosage increases. It can be seen that the reduction will tend to be flat. On the whole, as long as the starch is added, the water vapor of the film will permeate the performance will decrease, and changing the amount of starch will no longer have a greater impact on the water vapor permeability of the film.

(4) Film thickness analysis

**FIGURE 8:**
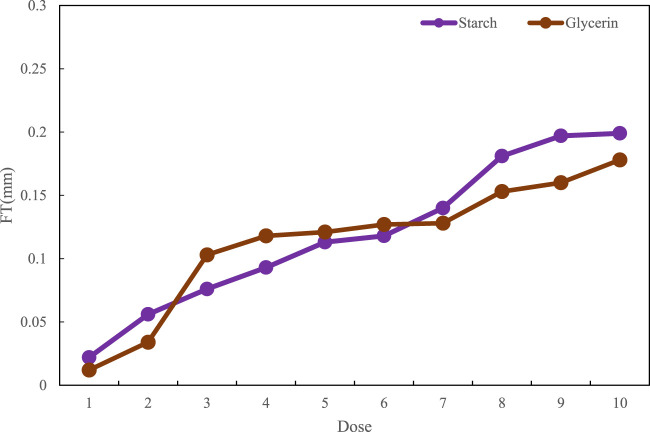
Film thickness analysis.

The thickness test of the film with different doses was carried out, and the thickness of the film under different doses was observed and tested. The detailed results are shown in Figure 8. With the increase in the quality of starch and glycerin, the film thickness and viscosity of the film also increase.

### 4.2 Stability analysis of the new nano-filled antibacterial layer packaging film

(1) Particle size, PDI and Zeta potential of the new nano-filled packaging film

The PDI obtained by different methods and methods represent different concepts. Generally, the PDI obtained by GPC represents the uniformity of molecular weight, that is, the normalized standard deviation of particle size. The detailed results are shown in Table 2.

**TABLE 2:** Particle size, PDI, Zeta potential index measurement results.

	1	2	3	4	Average
Particle size	23.17 nm	26.74 nm	23.66 nm	45.10 nm	29.67 nm
Zeta potential	−56.2 mv	−113.9 mv	−58.3 mv	−60.8 mv	−72.3 mv
PDI	−0.743	−0.79	−0.712	−0.646	−0.723

The smaller the PDI, it will closer to 0, the better the uniformity of the film, the larger the PDI, the closer to 1, the poorer the uniformity of the nano-film. It can be seen from the table that the nano-film prepared in this article has better uniformity.

Combining Table 2 and Table 3, it can be seen that the Zeta potential of the nano-film can indicate the excellent stability of the film.

**TABLE 3:** The relationship between Zeta potential and the stability of the dispersion system.

Zeta potential	Stability
0–5	Condensation
10–20	Poor
20–30	Generally
30–40	Quite
40–50	Good
Over 50	Excellent

**FIGURE 9:**
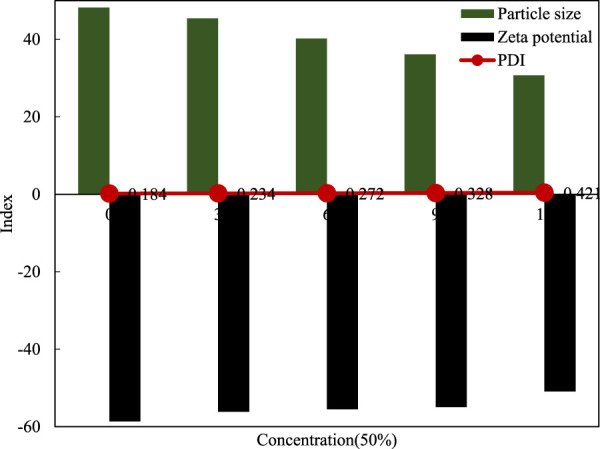
Long-term stability test of film.

The long-term stability test results of the new nano-filled antibacterial layer packaging film are shown in Figure 9. After a long-term stability test, after a change of time, the overall quality can still be maintained, and it will not appear until 9 months.

As time changes, PDI have a tendency to increase, the absolute value of Zeta potential decreases, and the particle size increases. The data shows that during storage, the particle size, dispersion uniformity and stability will deteriorate to a certain extent.

### 4.3 Antibacterial properties of packaging film on dairy products

Conduct antibacterial experiments on target microorganisms, including *S. aureus* and *E. coli*. The cryopreserved *S. aureus* and *E. coli* are sterilized by high temperature and then inoculated into a petri dish which has been pre-packed and passed through a high-temperature sterilization pot. After that, the activated bacteria liquid is stored in the petri dish.

The *E. coli* and *S. aureus* were diluted according to the multiple dilution method, and each multiple was made three in parallel, and the colony count results obtained are shown in Table 4 and Table 5.

**TABLE 4:** *Escherichia coli* dilution and total number of colonies.

Dilution	Number of colonies in different dilutions			Average number of colonies
	1	2	3	
10^3^	252	246	213	237
10^4^	22	24	26	24
10^5^	6	6	6	6

**TABLE 5:** *Staphylococcus aureus* dilution and total number of colonies.

Dilution	Number of colonies in different dilutions			Average number of colonies
	1	2	3	
10^3^	269	342	325	312
10^4^	82	84	80	83
10^5^	18	21	12	17

**FIGURE 10:**
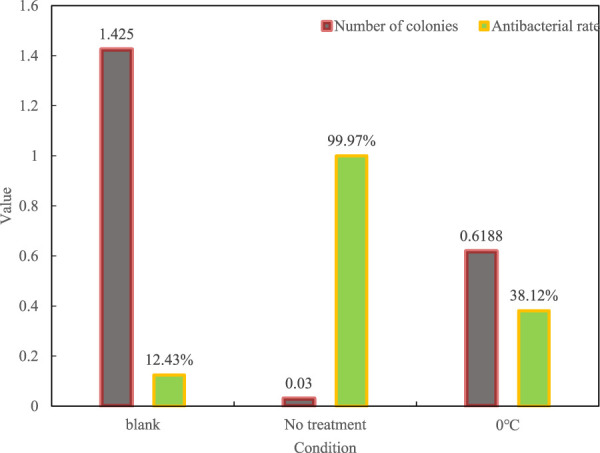
Antibacterial results after 120 days.

The antibacterial film prepared in this article was used for antibacterial treatment, and the number of colonies and the antibacterial effect under low temperature storage without antibacterial film treatment were compared. After 120 days of storage, the antibacterial results are shown in Figure 10.

It can be seen from the figure that after being placed for 120 days, the antibacterial rate of the untreated petri dish is only 12.43%, while the antibacterial efficiency of the untreated petri dish is as high as 99.97%, and the antibacterial rate under freezing is only 38.12%. It can be concluded that The antibacterial film has good thermal stability and high antibacterial properties.

### 4.4 Reverse recycling logistics of packaging

It is also important to recycle the new antibacterial layer packaging film used in dairy products. The disposal of packaging materials has led to a continuous increase in packaging material costs. Once a reverse packaging recycling system is established, network equipment recycling can reduce reliance on packaging materials, significantly reduce production costs, and reduce resource development. Reverse logistics is the reverse flow process of product transportation channels. The application of reverse logistics not only solves environmental problems, but also effectively relieves the pressure of resource shortage. The antibacterial packaging film produced in this article is recyclable, which can recycle materials, reduce resource waste, and reduce environmental pressure. Reverse logistics must fully understand market demand, maximize the use of value-added resources in waste, establish reverse logistics supply chain management, and achieve integrated growth.

The specific operation of reverse recycling logistics system is to establish a sound logistics network, including recycling points, sorting centers and reuse plants. These sites should be reasonably located in places easily accessible to consumers and equipped with appropriate equipment and technology for efficient disposal of recyclables.

Then a special recycling bucket is set up in the appropriate location to facilitate customers to recycle the discarded dairy packaging materials. At the same time, a clear recycling logo is added to the packaging to remind consumers to put the packaging into the recycling bucket.

The collected dairy packaging materials are sorted and sorted at the recycling point or at a dedicated sorting center. According to the type of packaging material, material and pollution level and other standards, it is classified as renewable and non-renewable materials.

Renewable dairy packaging materials should be sent to reuse plants for recycling processing.It is also necessary to establish an effective supervision and management mechanism to ensure the smooth operation of the entire reverse recycling logistics system.

## 5 Discussion

In the above experiments, it is verified that the packaging film prepared in this paper has high tensile strength, low light transmittance, low water vapor transmittance, and high stability and antibacterial property. These results show that the packaging film prepared in this paper will not affect the appearance and texture of dairy products, and can effectively inhibit the growth of bacteria, mold and other microorganisms, thereby reducing the risk of contamination in the packaging process of dairy products.

In conclusion, it is feasible to prepare the antibacterial layer packaging film of dairy products by using the new nano filling, and the performance is good. By using the new nano filling to prepare the antibacterial layer packaging film of dairy products, the safety and quality of dairy products can be effectively improved, its shelf life can be extended, and consumers’ requirements for food safety and quality can be met.

## 6 Conclusion

In this paper, a new type of nano-filled antibacterial layer packaging film for dairy products is prepared by extrusion casting method, and the mechanical properties and antibacterial properties of the film are experimentally analyzed. The results show that the antibacterial packaging film prepared in this paper has good mechanical properties and ensures the film’s permeability. The light rate and water vapor transmission rate, and the antibacterial properties of the packaging film are analyzed at the same time, and the antibacterial properties of the film are also guaranteed. In this article, the nano antibacterial starch film prepared with natural polymer materials such as sodium alginate and chitosan has good antibacterial properties, and also has good biodegradability, is safe and non-toxic, and makes up for the indispensability of traditional plastic products. It is degradable and has the shortcomings of potential safety hazards. It can be widely used in the food industry, biology and other fields, in line with the current people’s concept of environmental protection. More importantly, this article uses sodium alginate and chitosan to prepare drug-loaded nanospheres, which have antibacterial properties that can extend the shelf life of dairy products.

## Data Availability

The original contributions presented in the study are included in the article/Supplementary Material, further inquiries can be directed to the corresponding author.
